# Biochemical Characterization of the Tetrachlorobenzoquinone Reductase Involved in the Biodegradation of Pentachlorophenol

**DOI:** 10.3390/ijms9030198

**Published:** 2008-02-27

**Authors:** Lifeng Chen, Jian Yang

**Affiliations:** University of Saskatchewan, College of Pharmacy and Nutrition, 110 Science Place, Saskatoon, Saskatchewan S7N 5C9, Canada

**Keywords:** Biodegradation, enzyme, Yin-Yang regulation, homology modeling

## Abstract

Pentachlorophenol (PCP), a xenobiocide used to preserve lumbers, is a major environmental pollutant in North America. In spite of an expected high resistance to biodegradation, a number of aquatic and soil bacteria can degrade PCP. In this study, we cloned, expressed and purified tetrachlorobenzoquinone reductase (PcpD), the second enzyme in the PCP biodegradation pathway in *Sphingobium chlorophenolicum*. PcpD, present mainly as a homo-trimer, exhibited low but statistically significant activity in the reduction of tetrachlorobenzoquinone to tetrachlorohydroquinone. The optimal pH for PcpD activity was 7.0. PcpD was stimulated by tetrachlorohydroquinone at low concentrations but inhibited at high concentrations. Because of the constitutive expression and relatively high catalytic efficiency of downstream enzyme tetrachlorohydroquinone reductive dehalogenase, tetrachlorohydroquinone was unlikely to accumulate in high concentrations, suggesting that PcpD would only be stimulated by tetrachlorohydroquinone under *in vivo* conditions. It was also shown that PcpD was inhibited by PCP in a concentration-dependent manner. Therefore, PcpD was regulated by tetrachlorohydroquinone and PCP using a possible “Yin-Yang” mechanism, which maintained tetrachlorobeanzoquinone at a level that would neither significantly decrease the biodegradation of PCP nor cause cytotoxicity in *S. chlorophenolicum* cells. Structural model of PcpD showed that the putative tetrachlorobenzoquinone binding site, adjacent to the cofactor flavin mononucleotide and the 2Fe2S cluster, was situated in a deep pit on the surface and slightly positively charged.

## 1. Introduction

Pentachlorophenol (PCP) is a wide-spectrum xenobiocide that was widely used to treat lumbers before being banned by the US Environmental Protection Agency in 1987 [1–4]. It is highly toxic to both human and animals. Extensive exposure to PCP could cause cancer, acute pancreatitis, immunodeficiency and neurological disorders [5–8]. Because of its high toxicity and widespread distribution, PCP is currently listed as one of the major environmental contaminants in North America [9–12]. Although PCP is not a natural product and was expected to be highly resistant to biodegradation due to high and obstructive halogenation, a number of soil and aquatic bacteria, such as *Sphingobium chlorophenolicum* (*S. chlorophenolicum*), have evolved pathways to degrade PCP and use the ring-cleavage products of PCP as their carbon source [13–18].

**Figure 1.**
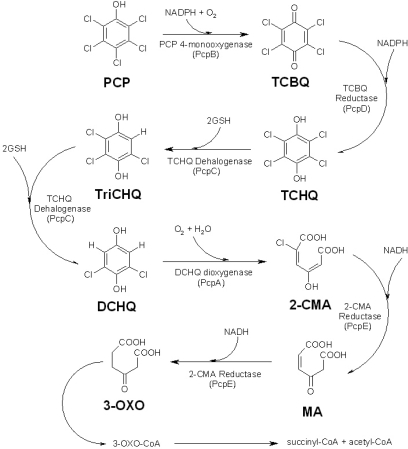
The pentachlorophenol (PCP) biodegradation pathway in *S. chlorophenolicum*. Pentachlorophenol, tetrachlorobenzoquinone, tetrachlorohydroquinone, trichlorohydroquinone, dichlorohydroquinone, 2-chloromaleylacetate, maleylacetate and 3-oxoadipate are represented by PCP, TCBQ, TCHQ, TriCHQ, DCHQ, 2-CMA, MA, and 3-OXO, respectively [13–15, 17–20, 25–29].

The PCP biodegradation pathway in *S. chlorophenolicum* strain ATCC 39723 contains five catalytic enzymes (Figure 1), which are evolved from at least two different metabolic pathways [13–14, 19]. This pathway is very inefficient both *in vitro* and *in vivo* [13, 19], mainly due to the low catalytic activity and substrate specificity of pentachlorophenol 4-monooxygenase (PcpB), which is the first and rate-limiting enzyme in the pathway [13–14, 19–20]. Early studies by Xun *et al*. showed that PcpB catalyzed the conversion of PCP to tetrachlorohydroquinone (TCHQ) using nicotinamide adenine dinucleotide phosphate (NADPH) as a co-substrate [13–14, 20]. However, Dai *et al*. recently reported that PcpB converted PCP into tetrachlorobenzoquinone (TCBQ) rather than TCHQ and the reduction of TCBQ to TCHQ was catalyzed by tetrachlorobenzoquinone reductase (PcpD) [19].

PcpD, originally deposited as a monooxygenase reductase in the GenBank, is encoded by gene *pcpD*, which is immediately downstream of gene *pcpB* encoding PcpB [19, 20]. It contains 324 amino acid residues and consists of three domains: an N-terminal NADPH-binding domain, a flavin-binding domain, and a C-terminal Rieske-type iron-sulfur cluster (2Fe2S) domain [19]. The proposed catalytic mechanism of PcpD is sequential electron transfer from NADPH to flavin mononucleotide (FMN) or flavin adenine dinucleotide (FAD), then from FMN or FAD to the 2Fe2S cluster, and finally from the 2Fe2S cluster to TCBQ [19]. In the current study, we cloned gene *pcpD* from *S. chlorophenolicum* strain ATCC 39723, overexpressed it in *Escherichia coli* (*E. coli*), and characterized the recombinant PcpD.

## 2. Results and Discussion

### 2.1 Cloning, expression and purification

**Figure 2.**
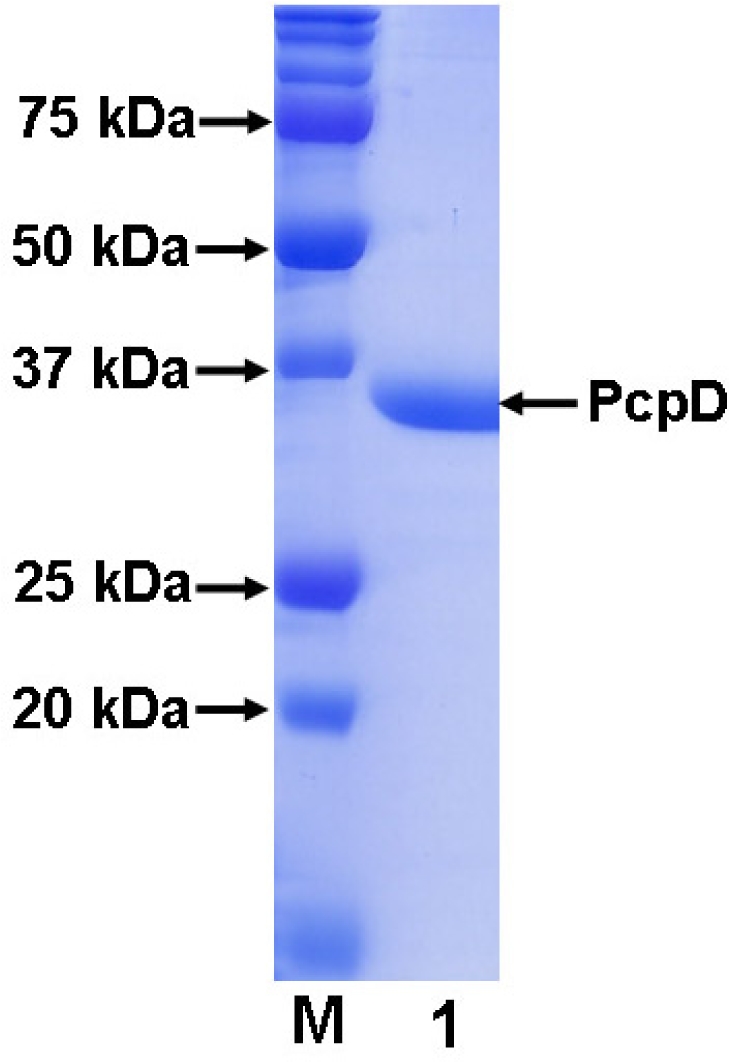
SDS-PAGE (12%) of purified His-tagged recombinant PcpD. Lane M was the protein standard.

As described below in the Materials and Methods section, gene *pcpD* was cloned by polymerase chain reaction (PCR) using the genomic DNA of *S. chlorophenolicum* strain ATCC 39723 as the template. The single PCR product was confirmed to contain 972 bp and share 100% sequence identity with gene *pcpD* by DNA sequencing. The pET30a(+) system was adopted to over-express C-terminal His-tagged recombinant PcpD in *E. coli*. Despite the high expression level, PcpD tended to form inclusion bodies. Extensive efforts, including varying the IPTG concentration, expression temperature, culture medium, expression vector and *E. coli* strain and co-transforming with chaperon vectors from the Takara Bio Inc., were attempted to overcome the problem without success. Although Dai *et al*. reported obtaining soluble PcpD by over-expressing gene *pcpD* in *Pseudomonas aeruginosa* (*P. aeruginosa*), the method yielded very little protein [19], indicating that either the protein expression level was very low or inclusion bodies were formed. This *P. aeruginosa* system could not be able to produce sufficient soluble protein for our biochemical and crystallographic studies. Since PcpD was discovered only recently and little is know about its biochemical properties, we decided to purify and characterize PcpD from the inclusion bodies in *E. coli* using a published protocol [21], while continuing to search for conditions to produce large quantities of soluble protein. To promote proper formation of the 2Fe2S cluster in PcpD during renaturing, we added 14 μM FeSO_4_ and 14 μM Na_2_S in the denaturing buffer. After refolding, PcpD was purified using a one-step affinity chromatography method on a 5 mL Ni-NTA agarose column. The Ni-NTA column bound PcpD was extensively washed with the wash buffer (>50 column volume) before PcpD was eluted with the elution buffer. The purified PcpD had higher than 95% purity as examined on a 12% SDS-PAGE gel (Figure 2). In addition, we observed that the extensive washing with the wash buffer was important for the purity of PcpD.

### 2.2 Physical properties

**Figure 3.**
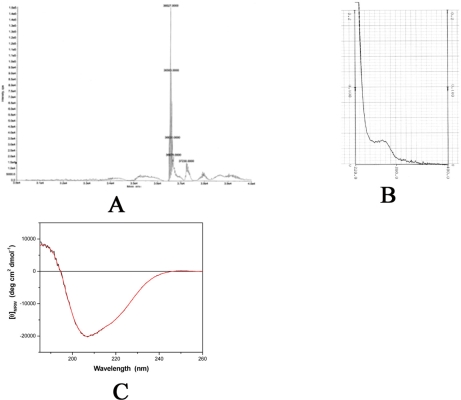
Physical properties of PcpD. (A). Mass spectrum (*m/z* range 30,000 – 40,000). (B). UV-visible spectrum (220–400 nm). (C). Raw (black) and smoothed (red) far-UV circular dichroism spectra (185–260 nm).

The molecular mass of the His-tagged recombinant PcpD was determined to be 36 kD by SDS-PAGE (Figure 2) and 36.5 kD by mass spectroscopy (Figure 3A). Both measurements were consistent with the calculated theoretical molecular mass of 36.5 kD. Because proteins purified from inclusion bodies are usually heterogeneous (21), the polymorphorism of PcpD was investigated by dynamic light scattering (DLS). PcpD existed as trimers (∼ 45% by mass) and aggregates of trimers, implying that the homo-trimer might be the physiological form for PcpD under *in vivo* conditions. Iron measurement by the phenanthroline method showed that 1 mole of PcpD contained 1.7 moles of iron. Thus, the 2Fe2S cluster was properly formed in PcpD, although the formation was not 100%. As shown in Figure 3B, the UV-visible spectrum of PcpD did not show the characteristic absorption of oxidized flavin mononucleotide (FMN) at 380 nm. In addition, the purified PcpD was colorless. This indicated that cofactor FMN or FAD was likely lost in the denaturing step during purification. To further examine whether PcpD was properly refolded, its secondary structure was determined by far-UV circular dichroism (Figure 3C). The refolded PcpD contained 33.2% β-strands and 16.4% α-helices. This was consistent with the study results of the secondary structure prediction (data unpublished) and the homology modeling described below in the structural model of PcpD and putative TCBQ binding site section. Therefore, we concluded that PcpD was accurately refolded and should be biologically functional.

### 2.3 Enzyme assay

**Figure 4.**
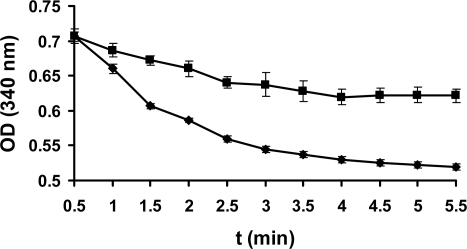
Enzyme assay of PcpD. The reaction mixture (200 μL) contained 50 mM phosphate buffer, pH 7.0, 150 μM TCBQ, 1.5 μM FAD, 240 μM NADPH, and 7 μg PcpD. As a blank control, boiled PcpD sample was added. The blank control and the PcpD reaction are shown as diamonds and squares, respectively.

The maximal absorption was at 293 nm and 281 nm for TCBQ and TCHQ, respectively, from UV-visible spectra collected on an Agilent 8453 diode array system. Neither TCBQ nor TCHQ would interfere with the characteristic absorption of NADPH at 340 nm. Therefore, the enzyme activity of PcpD was assayed by monitoring the UV absorption of the reaction system at 340 nm. As shown in Figure 4, PcpD indeed possessed low but statistically significant activity against TCBQ (P<0.0001 in unpaired student t-test at 95% significance level). The specific activity of PcpD under initial reaction conditions was 2.1 μmol/min/μg, which was almost twice of that for the PcpD over-expressed in *P. aeruginosa* [19]. Due to technical difficulties with the solubility for both TCBQ and PcpD, the kinetic parameters *k_cat_* and *K_m_* of PcpD could not be measured because the plot of the reaction rate (v) *versus* [TCBQ] was linear under the experimentally allowed TCBQ concentrations. Therefore, we could not identify whether PcpD followed *Michaelis–Menten* or allosteric kinetics. The observed reaction rate constant (*k_obs_*) was determined to be 0.094 min^−1^ and 0.032 min^−1^ for the reactions with and without PcpD, respectively. The catalytic power (*k_cat_*/*k_non_*) of PcpD was estimated to be 2.9 based on the ratio between the observed rate constants. The low activity of PcpD was possibly due to two reasons. First, PcpD was likely to be in an early stage of evolution. Secondly, PcpD was purified from inclusion bodies. Enzymes purified from inclusion bodies usually exhibit lower catalytic activity [22]. In addition, we observed that TCBQ reacted non-enzymatically with NADPH in the blank control. This raised an important question on whether the electron transfer catalyzed by PcpD was directly from NADPH to TCBQ or through the NADPH-FAD-2Fe2S-TCBQ chain. Further biochemical and structural studies are required to fully understand the catalytic mechanism of PcpD.

### 2.4 Effect of pH

**Figure 5.**
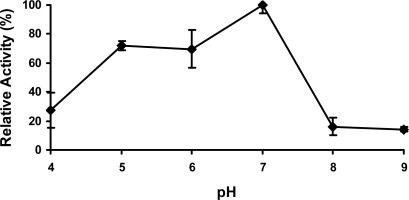
The effect of pH on PcpD activity. The reaction mixture (1 mL) contained 30 μg/mL PcpD, 1.5 μM FAD, 60 μM TCBQ, and 150 μM NADPH in 20 mM of the following buffers: acetate buffer, pH 4.0; acetate buffer, pH 5.0; phosphate buffer, pH 6.0; Tris-HCl buffer, pH 7.0; Tris-HCl buffer, pH 8.0; and TAPS buffer, pH 9.0. Boiled PcpD sample was used as a blank control under each pH.

The growth and biological function of soil bacteria are significantly influenced by soil pH. Previous studies show the optimal pH to grow *S. chlorophenolicum* is between 7.0 and 7.4 [23], implying the enzymes involved in the PCP biodegradation might work optimally at neutral pH. As we expected, the optimal pH for PcpD enzyme activity was indeed around 7.0 (Figure 5). PcpD was less sensitive to acidic pH than basic pH, maintaining more than 70% of its optimal activity even at pH 5.0. However, PcpD lost its activity quickly in basic solutions, retaining less than 20% of its optimal activity at pH 8.0. Two possible reasons might contribute to the rapid loss of activity for PcpD under basic pH. First, the OH^−^ anions changed the coordination geometries of the iron atoms in the 2Fe2S cluster, reducing the electron transfer of the 2Fe2S cluster. Secondly, the OH^−^ anions decreased the positive electrostatic potential at the active site (data unpublished), resulting in decreased binding of TCBQ. Studies by electron paramagnetic resonance (EPR) spectroscopy have been initiated to identify the catalytic mechanism of PcpD and clarify the reason for its rapid loss of activity in a basic environment.

### 2.5 The activity of PcpD was stimulated by TCHQ at low concentrations

**Figure 6.**
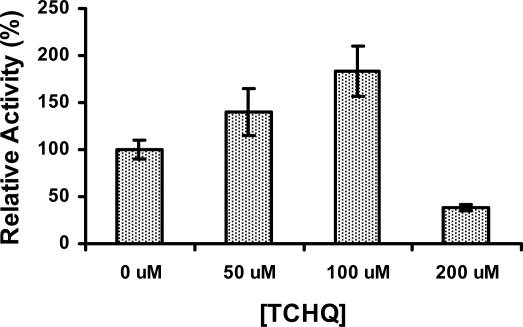
The stimulation effect of TCHQ on PcpD. The reaction mixture (200 μL) contained 50 mM phosphate buffer, pH 7.0, 150 μM TCBQ, 1.5 μM FAD, 240 μM NADPH, 7 μg PcpD and TCHQ. The concentration of TCHQ was at 0 μM, 50 μM, 100 μM, and 200 μM, respectively. Boiled PcpD sample was used as a blank control. The activity of PcpD under different TCHQ concentrations was compared to the activity in the absence of TCHQ, which was assigned a relative activity of 100%.

Since product inhibition is a common regulating mechanism observed in enzymes, we examined whether PcpD could be inhibited by its catalytic product TCHQ. Surprisingly, the activity of PcpD was stimulated by TCHQ at low concentrations but inhibited at high concentrations (Figure 6). The activity of PcpD was increased by 40% and 83%, respectively, when TCHQ was at 50 μM and 100 μM in reaction mixture. However, PcpD was inhibited by almost 60% when the concentration of TCHQ reached 200 μM. The stimulation of PcpD by TCHQ at low concentrations was possibly through the positive cooperative binding of the substrate TCHQ. Because positive cooperative binding of substrate is usually observed in multimeric enzymes and through allosteric effects [24], the positive cooperative binding of TCBQ to trimeric PcpD might also be caused by an allosteric effect. We hypothesized that the other monomer units in the PcpD homo-trimer underwent conformational changes to facilitate the binding of TCBQ molecules upon the formation of a TCHQ molecule at the active site of a monomer unit. Calorimetric titration and far-UV CD studies were undertaken to examine the above hypothesis. However, we could not eliminate the possibility that TCHQ reduced the aggregates of PcpD, which, in turn, increased the activity of PcpD. DLS studies would be carried out on PcpD samples before and after the reaction to confirm this possibility. The inhibition of PcpD by TCHQ at high concentrations was likely due to product inhibition. We suspected that the inhibition of PcpD by TCHQ might not happen in *S. chlorophenolicum* cells because TCHQ was unlikely to accumulate to high concentrations due to the constitutive expression and relatively high catalytic efficiency of the downstream enzyme TCHQ reductive dehalogenase (PcpC) [25–27]. Therefore, PcpD would only be stimulated by TCHQ under *in vivo* conditions. The biological significance of this stimulating effect on PcpD by TCHQ is to minimize the presence and reduce the toxicity of TCBQ in *S. chlorophenolicum*.

### 2.6 The activity of PcpD was inhibited by PCP

**Figure 7.**
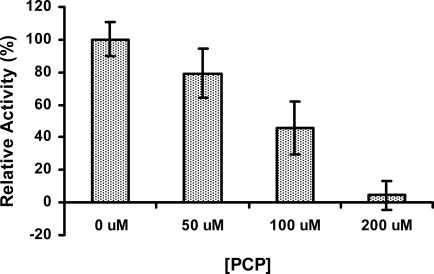
The inhibitory effect of PCP on PcpD. The reaction mixture (200 μL) contained 50 μM phosphate buffer, pH 7.0, 150 μM TCBQ, 1.5 μM FAD, 240 μM NADPH, 7 μg PcpD and PCP. The concentration of PCP was at 0 μM, 50 μM, 100 μM, and 200 μM, respectively. Boiled PcpD sample was used as a blank control. The activity of PcpD under different PCP concentrations was compared to the activity in the absence of PCP, which was assigned a relative activity of 100%.

Due to the inefficiency of PcpB [13–14, 19, 28–29], PCP may accumulate in high concentrations within the *S. chlorophenolicum* cells, and therefore any downstream catalytic reaction in the PCP biodegradation pathway would be affected by PCP. We investigated the effects of PCP on the reductase activity of PcpD. As shown in Figure 7, PcpD was inhibited by PCP in a concentration-dependent manner. PcpD lost approximately 20%, 55% and 95% of activity, respectively, when PCP was added to the reaction mixture at 50 μM, 100 μM and 200 μM. Furthermore, because the activity of PcpD was measured by monitoring the consumption of NADPH, if the nucleophilic PCP reacted with the electrophilic TCBQ to form complex molecules, the concentration of TCBQ in the reaction system would decrease, as would the usage of NADPH. We incubated a mixture of 100 μM PCP and 100 μM TCBQ at room temperature for 30 min and did not detect the formation of any complex molecule by HPLC (data unpublished). Thus, the inhibition of PcpD was due to competitive binding of PCP to the active site of PcpD. From the above experiments, we concluded that the activity of PcpD is likely to be regulated in a “Yin-Yang” fashion (*i.e*., between two opposing forces) by TCHQ and PCP. Through this “Yin-Yang” regulation, TCBQ might be controlled to a concentration *in vivo* that would neither significantly decrease the biodegradation of PCP by inhibiting the rate-limiting enzyme PcpB [19] nor cause cytotoxicity to *S. chlorophenolicum* cells.

### 2.7 Structural model of PcpD and putative TCBQ binding pocket

**Figure 8.**
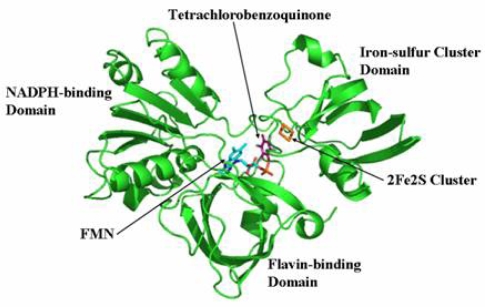
Ribbon representation of the structural model of PcpD. FMN, TCBQ and the 2Fe2S cluster are shown in stick models.

**Figure 9.**
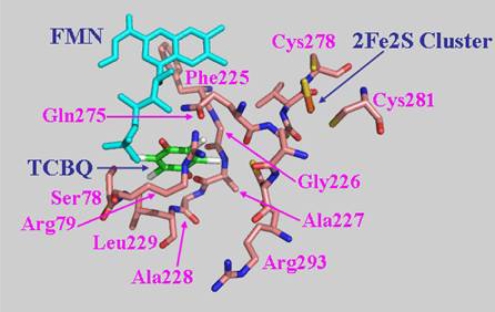
The putative TCBQ binding pocket. TCBQ was docked into the active site using AutoDock [31]. Residues forming the TCBQ binding pocket, the cofactor FMN and the docked TCBQ molecule are shown in stick models.

The biological function of a protein is determined by its three-dimensional structure. Since the crystal structure of PcpD has not been determined yet, we built a structural model of PcpD by homology modeling using the coordinates of phthalate dioxygenase reductase as the search model [30]. Based on the structural model, PcpD contained 37% β-strands and 16% α-helices, which was consistent with the far-UV circular dichroism study. Therefore, the structural model of PcpD was accurate and should be helpful in elucidating the biological function of PcpD. As shown in Figure 8, PcpD consisted of three domains. The active site, which was slightly positively charged (data unpublished), was situated in a deep pit on the surface. It was formed by residues from all three domains. TCBQ was docked into the active site of PcpD using AutoDock [31]. The putative TCBQ binding pocket was adjacent to the cofactor flavin mononucleotide (FMN) and the 2Fe2S cluster (Figure 9). It was formed mainly by residues Ser78, Arg79, Phe225, Gly226, Ala227, Ala228, Leu229, Gln275 and the cofactor FMN. The side-chain of Arg79 formed π-*p* interactions with the benzoquinone ring of TCBQ. The phosphate group of cofactor FMN, together with the side-chain of Gln275, interacted with one of the oxygen atoms of TCBQ through hydrogen bonds and helped in orienting TCBQ in its binding pocket. Proper orientation of TCBQ might be important for the electron transfer from the 2Fe2S cluster to TCBQ. However, the docked TCBQ molecule was about 8 Å from the 2Fe2S cluster, which was too far away for electron transfer. We suspected that PcpD might undergo conformational changes (such as domain movements) to bring the TCBQ molecule and the 2Fe2S cluster closer during the catalysis. Further protein crystallographic investigations are required to understand the catalytic mechanism of PcpD.

## 3. Experimental Section

### 3.1 Bacterial strains, expression vector and culture conditions

*S. chlorophenolicum* strain ATCC 39723 (American Type Culture Collection, VA, USA) was grown at 30 °C in 1 L of mineral media (K_2_HPO_4_ 0.65 g, KH_2_PO_4_ 0.19 g, MgSO_4_ 0.1 g, glutamic acid 4.0 g, NaCl 2.0 g, and FeSO_4_ 3 mg) for 4 d after inoculation with 1 mL of freshly grown cells. *Escherichia coli* strains DH5α and BL21-AI (Invitrogen, ON, Canada) were used as hosts for cloning and protein expression, respectively. Both strains were cultured at 37 °C in Lurie-Bertani (LB) media with 30 μg/mL kanamycin. Vector pET30a(+) (EMD Biosciences, CA, USA) was used as the protein expression vector.

### 3.2 Cloning and expression of gene pcpD

Genomic DNA of *S. chlorophenolicum* strain ATCC 39723 was extracted using a DNA extraction kit (Qiagen, ON, Canada). Using the genomic DNA as the template and a pair of DNA primers (forward: 5′-GGAGACCCGTCATATGACAAACCCCGT-3′ with an *Nde*I restriction site; backward: 5′-GTCGATCTCGAGGATGTCCAGCACCA-3′ with an *Xho*I restriction site) with sequences corresponding to the 5' and 3' ends of gene *pcpD*, respectively, we amplified a DNA fragment of the expected size by PCR reaction on an Eppendorf^®^ Mastercycler™ personal thermocycler (Eppendorf, ON, Canada). The PCR product was first confirmed to be gene *pcpD* be DNA sequencing and then ligated to the protein expression vector pET30a(+). The resulting plasmid pET/PcpD was transformed into *E. coli* DH5α cells by heat shock. Plasmid DNA was extracted from positive clones on LB-agar cultural plates containing 30 mg/mL kanamycin and transformed into *E. coli* BL21-AI cells. Protein expression in BL21-AI cells was induced by isopropyl β-D-1-thiogalactopyranoside (IPTG, 1 mM; final concentration) and L(+)-arabinose (0.2%; final concentration). Cell growth was allowed to proceed for 4 h after induction. The cells were harvested by centrifugation at 5,000 g for 20 min and the pellets were stored at −80 °C.

### 3.3 Purification of His-tagged recombinant PcpD

The recombinant PcpD was purified at 4 °C using a published protocol for purifying iron-sulfur cluster containing enzymes from inclusion bodies [21]. Briefly, a frozen pellet from 1 L cell culture was suspended in 40 mL of the lysis buffer (50 mM Tris-HCl, pH 8.0, 25% sucrose, 0.5 mM PMSF, 1 μM pepstatin A and 40 mg of lysozyme) and incubated with gentle rotation for 30 min. The cells were disrupted by sonication using a Sonifier^™^ 150 sonicator. The cell lysate was centrifuged at 20,000 g for 30 min. The supernatant was discarded and the pellet was washed once with 40 mL of the inclusion body buffer 1 (20 mM Tris-HCl, pH 8.0, 0.2 M NaCl, and 1% sodium deoxycholate) and four times with 40 mL of the inclusion body buffer 2 (10 mM Tris-HCl, pH 8.0, and 0.25% sodium deoxycholate). After washing, the pellet was completely resuspended in 22.5 mL of the denaturing buffer (10 mM Tris-HCl, pH 8.0, 8 M urea, 14 μM FeSO_4_, 14 μM Na_2_S, 0.5 mM PMSF, 1 μM pepstatin A and 0.1 mM NaN_3_). PcpD was renatured by adding 7.5 mL of the renaturing buffer (10 mM Tris-HCl, pH 8.0, 0.5 mM PMSF, 1 μM pepstatin A, and 0.1 mM NaN_3_) to the denaturing solution. The solution containing refolded PcpD was centrifuged at 20,000 g for 30 min. The supernatant was mixed with 5 mL of Ni-NTA agarose media and shaken for 2 h. The Ni-NTA agarose media were packed into a column and washed thoroughly with the wash buffer (50 mM phosphate buffer, pH 7.7, 0.3 M NaCl, 50 mM imidazole, 0.03% Triton-X 100, 0.5 mM PMSF, and 1 μM pepstatin A) at a flow rate of 2 mL/min until the elute UV absorbance was approximately zero. PcpD was then eluted with 20 mL of the elution buffer (50 mM phosphate buffer, pH 7.7, 0.3 M NaCl, 250 mM imidazole, 0.5 mM PMSF, and 1 μM pepstatin A). The purity of PcpD in the elution fractions was examined on a 12% SDS-PAGE gel. Fractions containing pure PcpD were combined, buffer-exchanged to storing buffer (10 mM PBS, pH 7.7, 0.5 mM PMSF), concentrated to 3 mg/mL, and stored at 4 °C.

### 3.4 Mass spectrometry

The PcpD sample (1 mg/mL in pure deionized water) was submitted for MALDI-TOF analysis on an API Qstar^®^ XL pulsar hybrid LC/MS/MS system (Applied Biosystems, USA) in the Saskatchewan Structural Sciences Centre. Positive ion mass spectra were acquired in the *m/z* range of 30,000 – 40,000.

### 3.5 Dynamic light scattering (DLS)

The PcpD sample (1 mg/mL in 10 mM sodium phosphate buffer, pH 7.7) was analyzed on a DynaPro-MS800 dynamic light scattering instrument (Wyatt Technology Corporation, CA, USA) in the Saskatchewan Structure Sciences Centre. 50 μL of the sample was loaded into a syringe equipped with a micro filter unit. After expelling 20 μL of dead volume into a waste container, the sample was slowly injected into a DLS cuvette. The DLS cuvette was inserted into the Micro Sampler of the instrument pre-calibrated with clean water. Twenty measurements were collected and the acceptable measurements were analyzed using software Dynamics V5.26.60 with the regularization algorithm.

### 3.6 UV absorption

The UV absorption spectrum of PcpD was recorded on a Shimadzu UV-265 UV-visible spectrophotometer (Shimadzu Scientific Instruments, MD, USA) at 25 °C using a 1 mL quartz cuvette of 10 mm path length. The sample concentration was 10 μg/mL in 10 mM sodium phosphate buffer, pH 7.7. The UV absorption spectrum was baseline corrected with the buffer.

### 3.7 Circular dichroism (CD)

Far-UV CD spectra were measured on a π*-180 kinetic circular dichroism spectrometer (Applied Photophysics, UK). The PcpD sample was prepared at 1 mg/mL in 10 mM phosphate buffer, pH 7.7, and examined in a 1 mm path length cell. The spectrometer was calibrated with D (+)-camphor sulfonic acid (CSA) at 192 and 290 nm. Six spectra were collected between 260 nm and 185 nm in 1 nm steps at 22 °C. The spectra were then averaged and smoothed using a 5-point Savitsky-Golay smoothing algorithm. The secondary structure was analyzed using CDNN version 2.1 [32].

### 3.8 Enzyme assay

All experiments in the current study were carried out in triplicate. The activity of refolded recombinant PcpD was assayed by monitoring the decrease of UV absorption at 340 nm using a UV-visible transparent 96-well plate. The experimental temperature was 25 °C. The reaction mixture (200 μL; final volume) contained 50 mM phosphate buffer, pH 7.0, 7 μg PcpD (boiled PcpD sample in the blank control), 150 μM TCBQ, 1.5 μM FAD and 240 μM NADPH. The UV absorption was taken at 0.5 min intervals for 5 min on a Synergy^™^ HT microplate reader from Biotek Instruments Inc. (Winooski, VT, USA). The UV path length was measured to be 0.71 cm. The extinction coefficient of NADPH (6220 M^−1^cm^−1^) was used to calculate the specific activity of PcpD.

### 3.9 Effect of pH

The effect of pH on the activity of PcpD was evaluated by measuring the decrease in UV absorption at 340 nm over a period of 5 min using a 10mm path length quartz cuvette. The reaction system (1 mL; final volume) contained 30 μg/mL PcpD (boiled PcpD sample in the blank control), 1.5 μM FAD, 60 μM TCBQ, and 150 μM NADPH. The buffers used in the study were 20 mM acetate buffer, pH 4.0; 20 mM acetate buffer, pH 5.0; 20 mM phosphate buffer, pH 6.0; 20 mM Tris-HCl buffer, pH 7.0; 20 mM Tris-HCl buffer, pH 8.0; and 20 mM TAPS buffer, pH 9.0.

### 3.10 Effects of TCHQ and PCP

The effects of TCHQ or PCP on the activity of PcpD was determined using the same method described above in the enzyme assay section except adding TCHQ or PCP to the reaction system with final concentrations of 50 μM, 100 μM and 200 μM, respectively. The PcpD reaction was allowed to proceed for 2 min. The decrease of UV absorption at 340 nm for the reaction system without adding TCHQ or PCP was used as the standard in the relative activity comparisons.

### 3.11 Structural model of PcpD and docking of TCBQ

The template (phthalate dioxygenase reductase; PDB ID: 2PIA) for building the structural model of PcpD was obtained from the protein-protein BLAST (blastp) search against the Protein Data Bank (PDB) using the amino acid sequence of PcpD. Pair-wise comparative sequence alignment between PcpD and phthalate dioxygenase reductase was performed by ClustalW [33]. Ten PcpD initial models were built by MODELLER V8.1 [34] using default parameters. The geometry and energy criteria for each initial model were evaluated by PROCHECK [35], ProSa II [36] and Verify3D [37]. The model with best geometry and lowest energy was selected as the final structural model for PcpD. TCBQ was docked into the active site of PcpD using AutoDock3 [31].

## 4. Conclusion

In this study, we showed that PcpD possessed low but statistically significant reductase activity against TCBQ and was regulated by PCP and TCHQ using a possible “Yin-Yang” mechanism. Structural modeling suggested PcpD might need to undergo conformational changes to accomplish the electron transfer from the 2Fe2S cluster to TCBQ. Further kinetic, spectroscopic and crystallographic studies are required to fully understand the catalytic mechanism and biological function of PcpD.
